# Preparation and Performance Evaluation of a Temperature and Salt Resistant Hydrophobic Associative Weak Polymer Gel System

**DOI:** 10.3390/molecules28073125

**Published:** 2023-03-31

**Authors:** Jiqiang Zhi, Yikun Liu, Jinfeng Chen, Lifeng Bo, Guohui Qu, Nan Jiang, Weizhong He

**Affiliations:** 1 School of Petroleum Engineering, Northeast Petroleum University, Daqing 163318, China; 2 Key Laboratory of Enhanced Oil Recovery, Northeast Petroleum University, Ministry of Education, Daqing 163318, China; 3 School of Electrical Engineering & Information, Northeast Petroleum University, Daqing 163318, China

**Keywords:** temperature and salt tolerance, weak gel preparation, profile control, fluid flow direction, plugging performance

## Abstract

We targeted high-temperature and highly saline old oil fields, whose environmental conditions could be attributed to the significantly high heterogeneity cause by long-term water flooding. The Huabei Oilfield was chosen as the research object. We developed a hydrophobic functional monomer–polymer with temperature and salt resistance by introducing the temperature-resistant and salt-resistant monomer NVP and a hydrophobic functional monomer into the main chain for copolymerization. We used a crosslinking agent with phenolic resin to prepare a weak gel system that showed temperature and salt resistance and investigated its temperature and salt resistance, infective property, plugging performance, liquid flow ability, micropore throat migration, and plugging characteristics. The results obtained using the infrared spectroscopy technique revealed the successful preparation of the phenolic resin crosslinker. The weak gel exhibited good temperature and salt resistance when the polymer concentration was 2000 mg/L, the cohesion ratio was 1:1.5, the additive concentration was 2000 mg/L, the reservoir temperature was 120 °C, and the injected water salinity was 40,300.86 mg/L. The average viscosity retention rate of the 90-day weak gel reached more than 80% and its microstructure was examined. The coreflow experiment results revealed that the weak gel system was characterized by good infectivity. After plugging the weak gel, the effect on the direction of the liquid flow was evident and the flow rate of the low permeability layer increased to a maximum of 48.63% under conditions of varying permeability levels. A significant improvement in the water absorption profile was achieved. The plugging was carried out through a sand-filling pipe under varying permeability conditions and the pressure measuring points in the sand-filling pipe were sucessfully pressurized. The migration ability of the weak gel was good and the blocking rate was >85%.

## 1. Introduction

The formation of advantageous channels in deep reservoirs under conditions of long-term water flooding can result in inefficient and ineffective circulation, causing a decrease in oil production and a rapid rise in water cuts. Depth profile control technology is often used to improve the swept volume of injected liquid [1,2,3]. However, some oil fields have deep reservoirs and high temperatures are recorded [4,5,6,7]. The re-injection of the water produced by the oilfield after treatment results in high salinity. A polymer weak gel profile control system mainly consists of using polymer and a crosslinking agent to form a weak gel, to achieve profile control and water plugging by blocking the high-permeability channel, and to control the inefficient and ineffective circulation of the reservoir. In such cases, the conventional weak gel system (consisting of polyacrylamide units) may lose its adhesive ability and become unstable at high temperatures and high salt levels. The loss of stability can be attributed to the decomposition of the side group, main chain fracture, charge shielding, double electric layer compression, and ion complex formation [8,9,10,11,12,13]. Therefore, improving the molecular structure stability is crucial to enhance the temperature and salt resistance of the gel [14,15].

In view of the high temperature and high salinity of the reservoirs in the Huabei Oilfield, a new temperature-resistant and salt-resistant polymer weak gel system was developed. In this paper, acrylamide (AM) was used as the main polymer, acrylic acid (AA) functional monomer was used to effectively improve the solubility of the polymer, N-vinylpyrrolidone (NVP) was used as the temperature resistant functional monomer, CHM was used as the hydrophobic functional monomer, PAM_10_-X1 was used as the chain transfer agent, and NaOH aqueous solution was used to adjust the pH value to synthesize the polymer. A new type of phenolic resin crosslinker was prepared with bisphenol A and formaldehyde and crosslinked with the polymer solution to form a more compact three-dimensional network structure with the polymer, improving the temperature resistance and salt resistance of the gel system.

To address these issues, we designed and prepared a hydrophobic monomer and synthesized a temperature- and salt-resistant weak gel system exhibiting a spatial network structure using phenolic resin as the crosslinking agent [16,17]. We analyzed the gel formation ability, temperature and salt resistance, injection performance, liquid flow effect, plugging performance, micropore throat migration, and plugging characteristics of the thermal- and salt-resistant gel consisting of the hydrophobic monomer [18,19,20,21,22]. The results reported herein provide a platform to achieve profile control in weak gels under conditions of high temperatures and salinity.

## 2. Results and Discussion

### 2.1. Preparation and Structural Characterization of the Polymer and the Crosslinking Agent

#### 2.1.1. Selection of Monomers for the Synthesis of the Hydrophilic Main Chain Units

**Figure 1:**
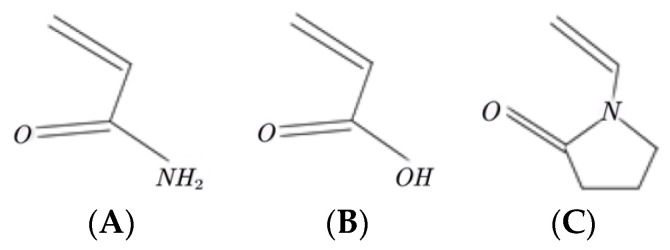
Structure of the main chain monomer. (**A**) AM (acrylamide); (**B**) AA (acrylic acid); (**C**) NVP (N-vinylpyrrolidone).

The primary factor affecting the viscosity and salt resistance of polymer solutions is the molecular structure of the polymers, as different structures exhibit different conformational features under different conditions. AM, AA, and NVP were chosen as the hydrophilic monomers to understand the temperature and salt resistance properties of the materials (Figure 1). AM offers excellent water solubility and high polymerization activity, while AA exhibits a high dissolution rate and strong thickening ability. NVP bears a five-membered heterocyclic structure in its side group, which provides excellent stability and reduces the likelihood of hydrolysis, thereby increasing the rigidity of the polymer chain and improving the thermal stability and viscosity of the polymer solution.

#### 2.1.2. Design and Preparation of the Hydrophobic Functional Monomer

**Figure 2:**
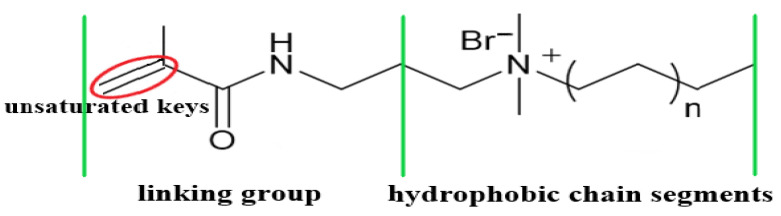
Schematic representation of the CHM structure of the hydrophobic monomer.

A hydrophobic monomer (CHM) was synthesized to cater to the high-temperature and high-salinity characteristics of oilfield reservoirs. As a water-soluble hydrophobic monomer (Figure 2), CHM enables micellar polymerization without the need for surfactants, thereby increasing the length of hydrophobic microblocks. Additionally, the large quaternary ammonium group present in the side group rigidifies the material, suppressing the process of polymer chain curling under high-temperature and high-salt conditions. This feature helps reduce the effect exerted by polymer curling on the properties of the hydrophobic micro-region.

**Figure 3:**
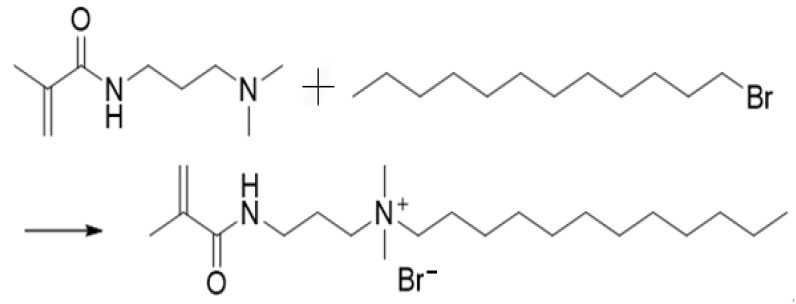
Synthetic route followed to synthesize CHM.

*N*-(3-dimethylaminopropyl) methylacrylamide (DMAPMA) (8 g, 0.04 mol), bromo-dodecane (12 g, 0.05 mol), and anhydrous acetone (35 mL) were taken in a three-way flask. All the reagents were added in an atmosphere of nitrogen. The mixture in the flask was heated using an oil bath and the solution was refluxed at 50 °C for 30 h. Following the completion of the reaction, the reaction mixture was evaporated using a rotary evaporator to obtain a light-yellow liquid. The obtained product was washed repeatedly with anhydrous ether to obtain a white precipitate. The precipitate was vacuum-dried at room temperature to obtain CHM. The synthetic route is shown in Figure 3.

**Figure 4:**
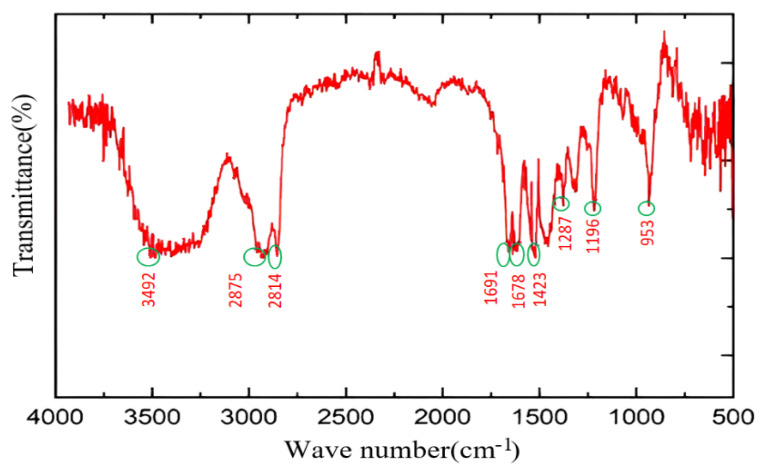
Fourier transform infrared (FTIR) spectral profiles recorded for CHM.

**Figure 5:**
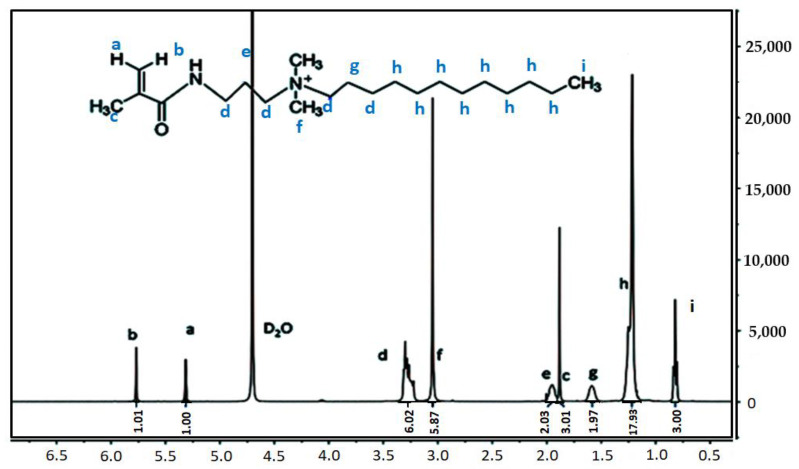
Nuclear magnetic resonance (NMR) profile recorded for CHM.

The hydrophobic monomer was characterized using the Fourier transform infrared (FTIR) and nuclear magnetic resonance (NMR) spectroscopy techniques, and the results are shown in Figure 4 and Figure 5. The FTIR profiles recorded for CHM reveals the presence of a peak at 3492 cm^−1,^ which can be attributed to N-H stretching. The peaks at 2875 and 2814 cm^−1^ represent the antisymmetric stretching and symmetric stretching vibrations of C-H. The peak at 1691 cm^−1^ represents the stretching of the olefinic double bond (C=C unit), the peak at 1678 cm^−1^ is attributed to the stretching vibration of the C=O (carboxyl) group, the peak at 1423 cm^−1^ reflects the presence of the N^+^ unit in the saturated C-H plane curved vibration region, the peaks at 1287 and 1196 cm^−1^ correspond to the telescopic vibration of the C-N and C-C units, respectively, and the peak at 953 cm^−1^ indicates the presence of the double substituted olefin unit. The NMR spectra recorded for the CHM unit were analyzed (i: 0.80–0.86 [t, 3H, CH_3_-CH_2_], h: 1.15–1.32 [d, 18H, CH_2_-(CH_2_)_9_-CH_3_], g: 1.50–1.62 [s, 2H, N-CH_2_-CH_2_-CH_2_], c: 1.86–1.92 [s, 3H, CH_3_-C(=CH_2_)(C=O)], e: 1.9–2.0 [s, 2H, N-CH_2_-CH_2_-CH_2_-N], f: 3.02–3.10 [s, 6H, CH_3_-N-CH_3_], d: 3.20–3.35 [m, 6H, CH_2_-N], a: 5.30–5.35 [s, 1H, H-C=C-CH_3_], b: 5.75–5.80 [s, 1H, H-C=C-C=O]. The presence of the characteristic peaks indicates the successful synthesis of the designed and targeted functional monomer.

#### 2.1.3. Preparation of the Hydrophobic associative Polymers

**Figure 6:**
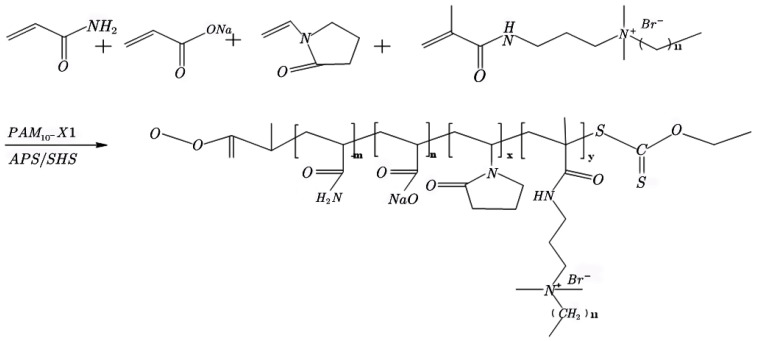
Reaction path followed to obtain the hydrophobic associative polymer.

AM, AA, NVP, CHM, and a chain transfer agent PAM_10_-X1 were added to the reactor. An aqueous solution of NaOH was used to adjust the pH of the solution to neutral following its dissolution in deionized water. The solution was thoroughly mixed. Subsequently, the mixture was placed in a reaction kettle and deoxygenated using nitrogen. Following this, the low-temperature REDOX initiator, ammonium persulfate, was added, along with the chain transfer agent sodium formate, to control the molecular weight of the polymer. The gelatinous material was removed after 6 h, and the polymer underwent primary and secondary granulation. Finally, the polymer was dried and broken to yield the hydrophobic associative polymer. The reaction path followed to obtain the final product is shown in Figure 6.

#### 2.1.4. Preparation of the Phenolic Resin Crosslinker

**Figure 7:**
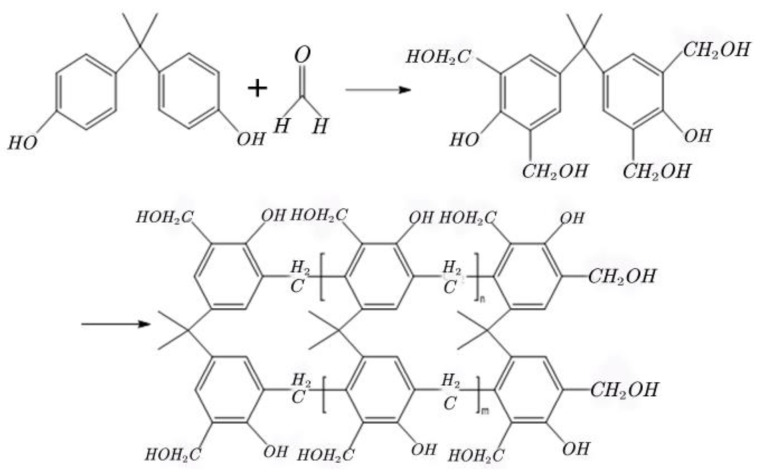
Synthetic route followed to prepare the crosslinker.

A certain amount of bisphenol A, the catalyst (6% sodium hydroxide solution), and a formaldehyde solution were added to a four-way flask equipped with a thermometer and a stirrer. The flask was mounted on a reflux device and attached to a constant-pressure liquid separation funnel. The mixture was heated and stirred to form a homogenous solution. Following this, the remaining catalyst was added to the mixture, the pH value of the solution was adjusted, and the temperature was allowed to rise. The reaction mixture was stirred and the crosslinker was obtained after cooling the reaction mixture following the completion of the reaction (Figure 7).

#### 2.1.5. Structural Characterization of the Crosslinker

**Figure 8:**
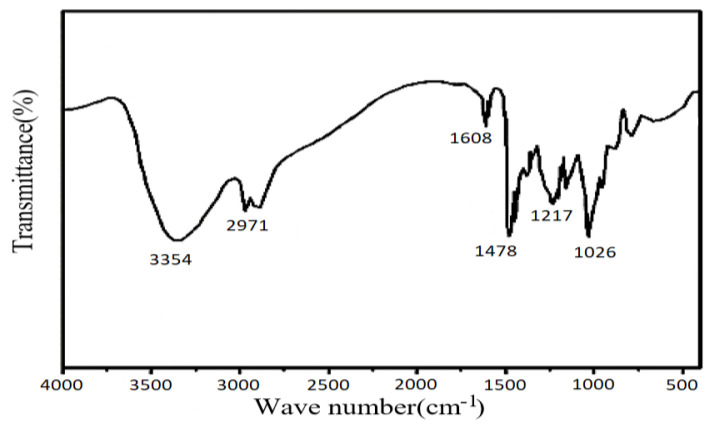
FTIR profiles recorded for the crosslinker.

Figure 8 presents the infrared spectral profile recorded for the phenolic resin-based crosslinker. The peak at 3354 cm^−1^ represents the stretching vibration of the intermolecular hydrogen bond, the peak at 2971 cm^−1^ can be attributed to the telescopic vibration of the -CH_2_^−^ unit, the peak at 1608 cm^−1^ is the skeleton vibration of the C=C unit in the benzene ring, the peak at 1478 cm^−1^ presents the skeleton stretching vibration of the benzene ring, and the peaks at 1217 and 1026 cm^−1^ correspond to the C-O stretching vibration of the CO unit in the hydroxymethyl group. The data reveal the successful synthesis of the phenolic resin-based crosslinking agent.

#### 2.1.6. Crosslinking Mechanism of the Hydrophobically Associating Polymer and the Phenolic Resin

**Figure 9:**
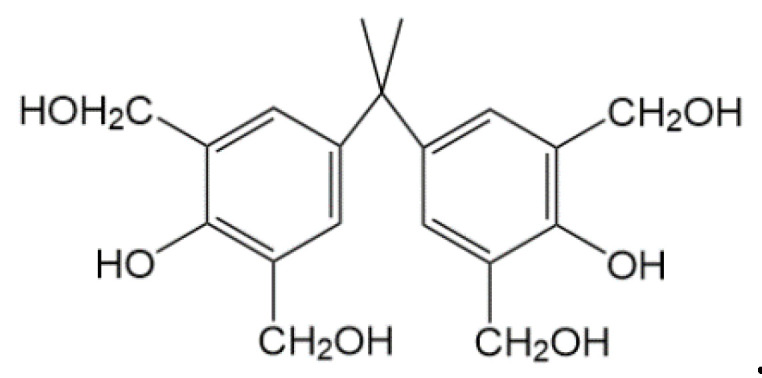
Molecular structural formula of hydroxymethyl (-CH_2_OH) in a novel phenolic resin crosslinking agent.

**Figure 10:**
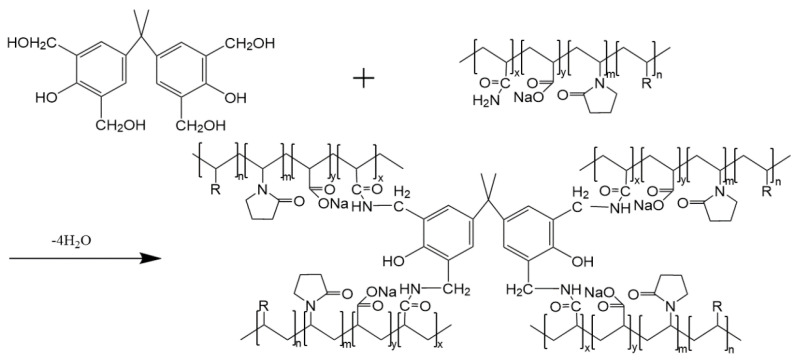
Schematic diagram of crosslinking mechanism between hydrophobic association polymer and phenolic resin.

The novel phenolic resin crosslinking agent is essentially a crosslinking agent made of bisphenol A and formaldehyde in a certain proportion, mainly through the condensation reaction between bisphenol A and formaldehyde to produce hydroxymethyl(-CH_2_OH). Its structural formula is as follows (Figure 9):

Hydroxymethyl (-CH_2_OH) of the benzene ring in the phenolic resin reacts with the amide group (-CONH_2_) in the polymer molecules to remove a molecule of H_2_O and form-CH_2_-NH-CO-, as it is shown in Figure 10 (R in the figure represents the hydrophobic functional monomer), finally forming a three-dimensional network structure. Compared with the conventional phenolic resin crosslinking agent, the introduction of bisphenol A introduces more benzene rings into the polymer and the weak gel system formed has better temperature resistance and stability.

### 2.2. Performance Evaluation of the Hydrophobic Associative Polymer Gels

#### 2.2.1. Influence of the Additive Concentration on the Gel Properties

The reinjection salinity was 40,300.86 mg/L and guaranteed the gel formation at 120 °C. The concentration of the fixed polymer was set at 2000 mg/L and the concentration of the crosslinker was set at 3000 mg/L while varying the concentration of the additive to 1500, 2000, and 2500 mg/L. The experimental results are presented in Table 1. As the concentration of the additive increased, the viscosity of the gel system gradually increased while the viscosity retention rate gradually decreased. A concentration of 2000 mg/L yielded a good viscosity retention rate and viscosity, indicating that it was the optimal concentration.

#### 2.2.2. Effect of Polymer and Crosslinker Concentration on Gel Properties

Table 2 presents the results obtained under conditions of varying polymer and crosslinker concentrations. The reinjection salinity was maintained at 40,300.86 mg/L and the temperature was maintained at 120 °C during the experiments. The polymer concentrations were varied to 1800, 2000, and 2250 mg/L and the polymerization ratio was in the range of 1:1.2–1:2. A polymer concentration of 1800 mg/L resulted in a poor viscosity retention rate and a final adhesive strength of <1000 mPa·s at 90 days. A polymer concentration of 2000 mg/L yielded a final gel forming strength of >1000 mPa·s and the maximum viscosity retention rate at 90 days was recorded when the cohesion ratio was 1:1.5. A polymer concentration of 2250 mg/L resulted in a low 90-day viscosity retention rate. This rate was lower than that recorded at a concentration of 2000 mg/L under conditions of the same cohesion ratio. The optimal conditions were determined based on the results obtained from the long-term aging experiments (polymer concentration: 2000 mg/L; crosslinking agent concentration: 3000 mg/L; crosslinking agent concentration: 2000 mg/L).

#### 2.2.3. Stability of the Hydrophobic Associative Polymers

**Figure 11:**
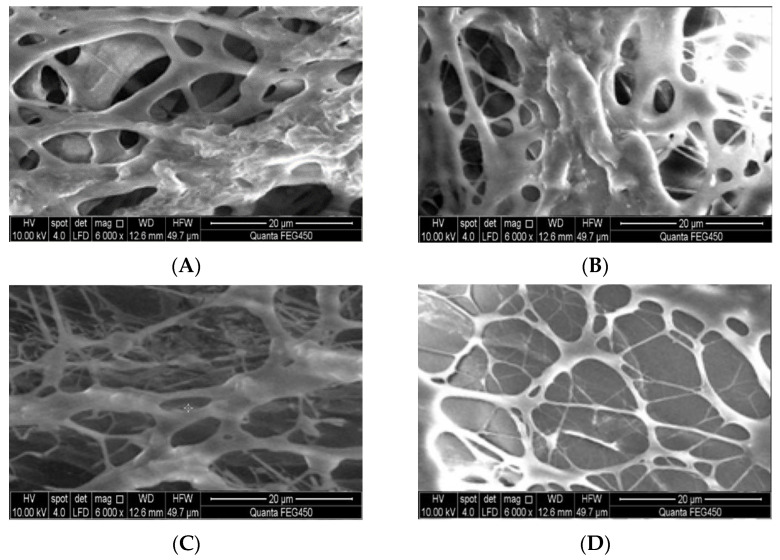
Gel microstructure recorded under conditions of different aging times. (**A**) Gel aging for 5 days; (**B**) Gel aging for 30 days; (**C**) Gel aging for 60 days;(**D**) Gel aging for 90 days.

A long-term aging process results in the thermal degradation of the polymer, which eventually results in gel instability. The optimal ratio for the hydrophobic associative polymer was determined at a polymer concentration of 2000 mg/L, a crosslinking ratio of 1:1.5, and a crosslinking agent concentration of 2000 mg/L. The data were recorded at a temperature of 120 °C and salinity of 40,300.86 mg/L with 5-, 60-, and 90-day-old gel samples. The microscopic morphology was observed using a scanning electron microscope, and Figure 11 presents the morphology of the gel under these conditions. A prominent spatial network is formed between 5 and 90 days. An increase in the aging time is accompanied by a decrease in the density of the network framework. The degree of smoothness and firmness of the untreated sample decreases over time. However, the network structure remains intact, maintaining a good spatial framework. The *N*-vinylpyrrolidone side groups on the molecular chains of the hydrophobic associative polymers enhance the rigidity of the polymers and the stability of the main chain, preventing the polymers from curling and hindering the process of thermal degradation. Depolymerization and the formation of random fractures under high-temperature and high-salinity conditions can be avoided under these conditions. This helps improve the stability of the weak gel post-gelling. The weak gel system prepared using the oilfield injection water maintains long-term stability under high-temperature conditions.

**Figure 12:**
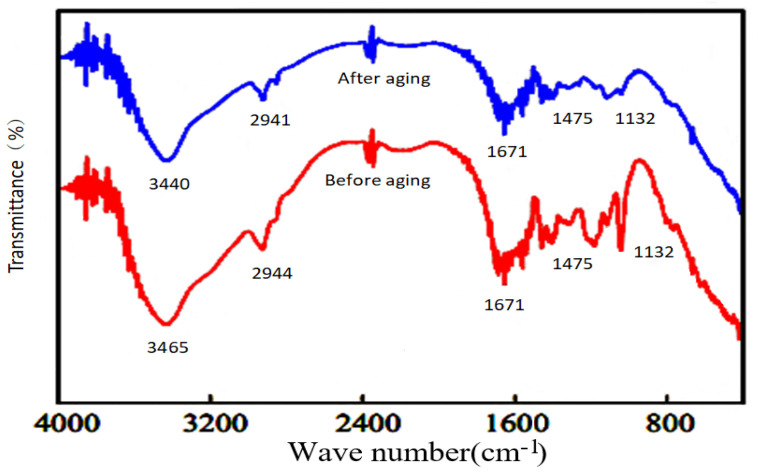
FTIR profiles recorded before and after aging.

The FTIR spectral profiles were recorded for the weak gel before and after aging (Figure 12). The peak positions changed significantly before and after aging. For example, the peak at 3448 cm^−1^ shifted to 3423 cm^−1^ after aging. The hydrolysis of the amide group (resulting in the formation of a carboxylic acid group) in the hydrophobic associative polymer resulted in the strengthening of the hydrogen bond. This resulted in a shift in the peak position towards the low wavenumber region. Analysis of the IR profile revealed that the hydrophobic associative polymer undergoes open-chain thermal degradation of the amide group. However, the microstructure of weak gels did not change significantly following high-temperature aging, indicating that the chain opening process associated with the hydrophobic associative polymer is not the primary thermal degradation mode that results in the destruction of the structure of the weak gel network. Hydrophobic associative polymer molecules undergo thermal degradation (polymerization or random fracture occurs), but the degree of degradation is relatively small. The five-member ring structure of NVP can effectively increase the steric hindrance, improve the rigidity of the molecular chain, inhibit the process of hydrolysis of amide groups to a certain extent, improve the thermal stability of the polymer backbone, and weaken the strength of the adverse effects generated under conditions of thermal degradation. The results revealed that the hydrophobic associative polymer gel system prepared using reinjection water retained its stability after long-term high-temperature aging. The rigidity of the three-dimensional network structure formed by the hydrophobic associative polymer and phenolic resin-based crosslinker can suppress the process of polymer chain curling (occurring under saline conditions) to a certain extent. The gel does not undergo dehydration shrinkage.

### 2.3. Evaluation of the Performance of the Hydrophobic Associative Polymer Gel

**Figure 13:**
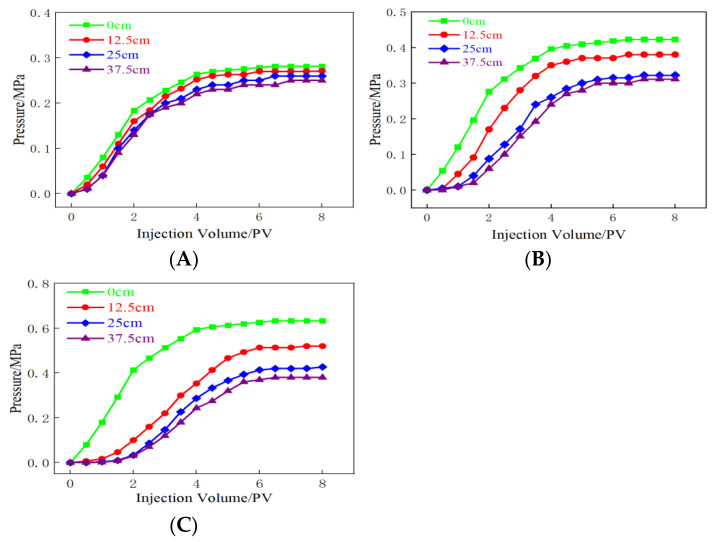
Injectivity of the gel system in sand-filled tubes characterized by different permeability lengths. (**A**) Permeability at 800 × 10^−3^ μm^2^; (**B**) permeability at 300 × 10^−3^ μm^2^; (**C**) permeability at 100 × 10^−3^ μm^2^.

Figure 13 presents the pressure curve recorded for the hydrophobic associative polymer gel system. The variation in the curve with changes in the injection amount was recorded under conditions of varying permeability. The sand-filling tube with high permeability was used for the experiments. Each pressure measuring point started up steadily, and stability was attained after 3 PV injections (Figure 13A). The pressure at the points that were 0 and 12.5 cm away from the injection point increased rapidly with a decrease in permeability. The rate of increase at these points was higher than the rate of increase in pressure recorded at the points that were 25 and 37.5 cm away from the injection point. The pressure at each measuring point became stable after the pressure successively started (Figure 13B). Figure 13C was 100 × 10^−3^ μm^2^. The pressure rises rapidly at the injection end, and an increase in the amount of injection volume results in an increase in pressure at the measuring point. It was observed that the pressure at each measuring point increased steadily when the sand-filled pipes characterized by varying degrees of permeability were used for the experiments. The hydrophobic associative polymer gel could be effectively injected into the system.

### 2.4. Direction of Liquid Flow Parallel to the Double Pipes

Different permeability levels were tested by conducting a double-tube parallel experiment when the reinjection salinity was 40,300.86 mg/L, and the temperature was 120 °C. Water flooding was achieved, and 0.3 PV of the polymer gel was injected into the system. The gel was aged for 5–7 days, and the instantaneous flow rate of the high- and low- permeability layers was recorded under conditions of water flooding. The objective was to determine whether the hydrophobic associative polymer gel could effectively plug heterogeneous layers while maintaining good flow characteristics. The rates before and after plugging under conditions of varying degrees of permeability were recorded (Table 3). The results revealed that fluids shifted from the hyperpermeability layer to the hypopermeability layer, resulting in a decrease in the hygroscopic layer and an increase in the hygroscopic layer when the gel was injected into the parallel core. A decrease in the core permeability resulted in a significant increase in the hygroscopic layer. These findings suggest that a gel profile control system can be adopted for the strata with strong heterogeneity to effectively plug the high-permeability layer [23,24,25].

**Figure 14:**
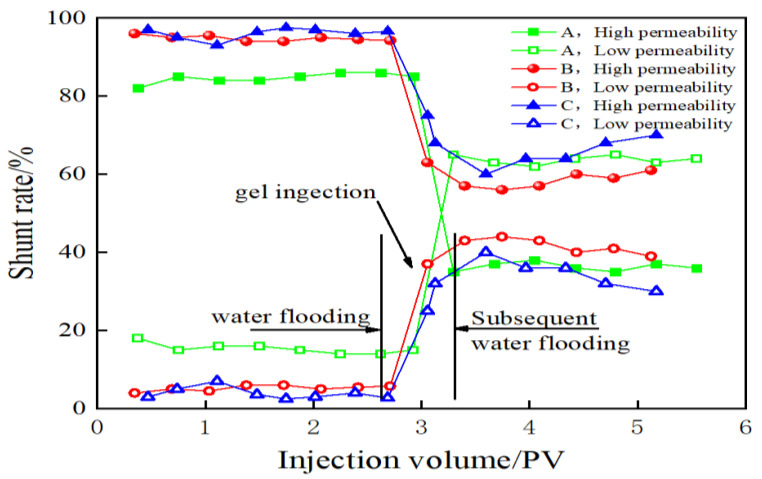
Variation in the flow rate of high- and low-permeability layers in cores with different permeability levels as a function of injection volume (A: Permeability ratio: 5; B: Permeability ratio: 40; C: Permeability ratio: 200).

In Figure 14, the graph illustrates how the diversion rate corresponding to high- and low-permeability layers varies with the injection amount under conditions of varying core permeability levels. The process of water flooding exerts good steering effects when the difference in permeability level is 5. It was also observed that the suction volume and utilization degree of the low permeability layer increased significantly. However, the extent of liquid imbibition achieved for the low-permeability layer increased when the permeability level difference was 40. The extent of increase recorded under these conditions was higher than the extent of increase recorded under conditions of the water flooding stage before plugging. However, the range of increase reduced significantly, and the liquid flow direction capacity decreased. An increase in the permeability level resulted in a decrease in the degree of utilization of the low permeability layer. This could be attributed to increased heterogeneity, and the strength of the effect of the direction of flow of the liquid was weakened.

### 2.5. Gel Plugging Performance

**Figure 15:**
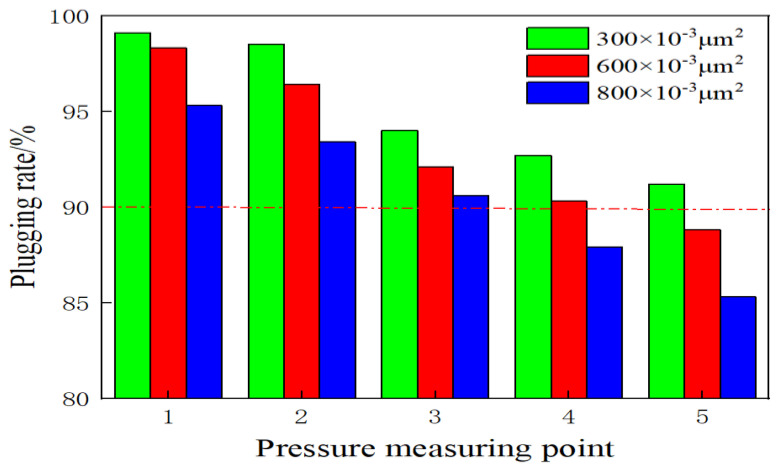
Change in the plugging rate at each measuring point of the sand-filled pipe exhibiting varying permeability characteristics.

Figure 15 presents the change in the plugging rate observed at each measuring point in the sand-filled pipe that exhibits varying permeability characteristics following the formation of the gel from the weak gel. The plugging rate at each measuring point was analyzed, and it was observed that the plugging rate gradually decreased as the measuring point moved away from measuring point 1. An increase in permeability resulted in the deterioration in the plugging conditions, and the conditions were poorer than the conditions recorded at the same pressure point. For instance, the plugging rate at each measuring point was >90% when the permeability was 371 × 10^−3^ μm^2^. Similarly, the plugging rate at measuring point 5 was 88.8% at a permeability of 680 × 10^−3^ μm^2^. The plugging rates at the pressure measuring points 4 and 5 were 87.9 and 85.3%, respectively, at a permeability of 805 × 10^−3^ μm^2^. The analysis of different permeability levels revealed that each measuring point was successfully and appropriately pressurized. The sealing rate was greater than 85%, suggesting that the gel exhibited excellent plugging performance.

### 2.6. Micropore-Throat Migration and Plugging Characteristics of the Gel System

**Figure 16:**
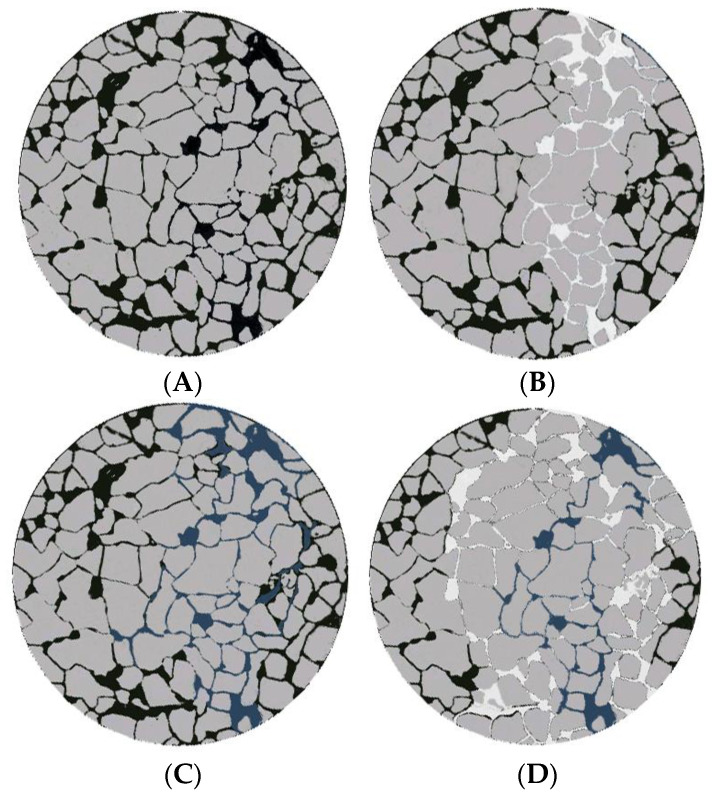
Schematic representation of the oil displacement effect produced by the microsimulation glass etching model. (**A**) Initial; (**B**) Water flooding; (**C**) Gel injection;(**D**) Subsequent water flooding.

The migration, retention, and deformation laws of the gel system were studied in a porous media using the micro-simulation glass etching model (Figure 16).

Once the micro-model became saturated with oil (Figure 16A), the system is water-flooded, causing the water to channel only along the hyperpermeability strip in the direction of the main stream line (Figure 16B). A high-water channeling speed was recorded. Unfortunately, the crude oil in the remaining areas of the model (which make up 72.4% of the total area) was not utilized. The use of the high-power microscopy technique revealed that the gel enters the large pores and moves forward under pressure, allowing the extensive utilization of oil (Figure 16C). The extent of gel coverage reached 43.6%.

Another water flooding process was carried out after a five-day-long model aging process (Figure 16D). It was observed that the gel successfully blocked the original large pores under these conditions. This change in flow direction displaced the direction of the non-mainstream line and the crude oil in the low permeability area, resulting in an expansion of the sweep area. This helped in the effective utilization of the remaining oil. As a result, the recovery rate reached 68.3%. In other words, an increase of 40.7% was achieved with respect to the initial recovery rate. Thus, the goal of improving the oil recovery rate was achieved.

## 3. Materials and Methods

### 3.1. Reagents and Instruments

Experimental agents: acrylamide (AM, crystal, ≥98%), Aladdin Biochemical Technology Co., LTD, Shanghai city, China; acrylic acid (AA), Aladdin Biochemical Technology Co., LTD, Shanghai city, China; *N*-vinylpyrrolidone (NVP), Aladdin Biochemical Technology Co., LTD, Shanghai city, China; sodium bisulfite, Kelon Reagent Co. LTD, Chengdu city, China; hydrophilic monomer (CHM), homemade; sodium formate, Mingguan Chemical Co. LTD, Jinan city; urea, Shengqiang Chemical Industry Co. LTD, Shandong province, China; phenolic resin crosslinking agent, homemade; crosslinking agent, homemade; ammonium persulfate, Kelon Reagent Co. LTD, Chengdu city, China.

Properties of the water used: salinity of the injection water used in the oilfield: 40,300.86 mg/L. Ion composition (mg/L): CO_3_^2−^ (125), HCO_3_^−^ (428.27), Cl^−^ (24,096.9), SO_4_^2−^ (3.22), Na^+^ (14,424.4), K^+^ (89.58), Ca^2+^ (998.1), Mg^2+^ (122.22), Fe^3+^ (12.72), and Al^3+^ (0.45).

Equipment used: electric blast constant temperature drying oven, Huitai Instrument Manufacturing Co., LTD, Shanghai city, China; Haake rheometer, Thermo Fisher Scientific, Karlsruhe, Germany; QuantaFEG 450 type Environmental Scanning Electron Microscope (SEM), FEI Company, Hillsboro city, OR, USA; Nicolet iS10 type infrared spectrometer, Thermo Nicolet Corporation, Madison city, WI, USA: HBYQ-2 high-temperature and high-pressure core flow test device, Huabao Petroleum Instrument Co., LTD, Yangzhou city, China; HBS300/50 double cylinder constant speed constant pressure pump, Huabao Petroleum Instrument Co., LTD, Yangzhou city, China; electronic balance (BS124 type), Sartorius Corporation, Göttingen, Germany; J-1 type enhanced electric Stirrer, Wanhe Instrument Co., LTD, Changzhou city, China; ZN-08 type laboratory shredder, Xingshi Lihe Technology Development Co. LTD, Beijing city, China; microsimulation glass etching model, homemade; OLYMPUS BX41 type optical microscope, microscope, Olympus Corporation, Tokyo city, Japan; micropump, Hai’an Petroleum Scientific Research Instrument Co. LTD, Nantong city, China.

### 3.2. Experimental Methods

(1) Preparation of the polymer and the crosslinking agent: AA, AM, NVP, CHM, chain transfer agent, and deionized water were mixed in beakers. An aqueous solution of NaOH was used to adjust the pH of the system. The samples were washed, dried, and crushed to obtain dried polymer powder. Bisphenol A and formaldehyde were used to prepare a new phenolic resin-based crosslinking agent. The molecular structure of the newly-developed phenolic resin contains more numbers of hydroxyl-methyl compared to the conventional phenolic resin prepared using phenol and formaldehyde. This allows for the formation of a more compact three-dimensional network structure with the polymer, leading to an improved temperature and salt resistance of the gel system.

(2) Evaluation of gel properties: the gel strength was measured by analyzing the gelatinization viscosity. The experiments were conducted at a temperature of 120 °C. The salinity of the injected water was 40,300.86 mg/L and the water salinity was measured over 90 days. The HAKKE rheometer was operated at 7.34 s^−1^. The adhesive viscosity of the material was monitored continuously to study the strength and stability of the system [26,27]. The average viscosity retention rate was calculated as follows:

$$\mathrm{Average}\;\mathrm{viscosity}\;\mathrm{retention}=\frac{\mathrm{Viscosity}\;\mathrm{of}\;\mathrm{gel}\;\mathrm{system}\;\mathrm{at}\;90\;\mathrm{days}}{\mathrm{Average}\;\mathrm{viscosity}\;\mathrm{within}\;90\;\mathrm{days}\;\mathrm{after}\;\mathrm{gel}\;\mathrm{formation}}\times 100\%$$

(3) Scanning electron microscopy: the QuantaFEG 450 environmental scanning electron microscope was used to observe the changes in the microstructure of the gel. The SEM images were recorded at different aging times under conditions of constant temperature (120 °C) and injected water salinity (40,300.86 mg/L). A certain amount of the samples was placed in liquid nitrogen during the pretreatment stage of the polymer gel samples to freeze them. This process ensured that the structure of the polymer gel remained unaffected during the experiments. The samples were freeze-dried for 24 h, following which the polymer gel sample was attached to the surface of a copper test plate using conductive adhesive. Finally, powdered gold was sprayed onto the surface of the freeze-dried polymer gel sample to improve the conductivity of the gel sample [28,29].

(4) Evaluation of the injection performance: a sand-filled tube (Φ 2.5 × 50 cm) and three pressure measuring points were used to inject the gel solution at a constant rate (0.3 mL/min). The pressure changes at each pressure point were recorded to study the injection performance of the solution of the hydrophobic polymer gel.

(5) Direction of the parallel flow in a double pipe: an artificial core (permeability: Φ 2.5 × 10 cm) was used to conduct the experiment and record the pressure changes at the injection point to investigate the blocking effect exerted by the polymer gel system under conditions of high temperature and high core content. The changes in seepage properties that occurred before and after plugging were recorded.

(6) Weak gel plugging performance: the core flow experiment was conducted at a formation temperature of 120 °C using a long sand-filled tube measuring Φ 3.8 × 100 cm and equipped with four pressure measuring points. Initially, the water flooding was achieved at a constant speed of 0.3 mL/min, and the increase in pressure was recorded at each pressure measuring point of the sand-filled tube. The process of water flooding was conducted at the same speed following the completion of the aging process, and the permeability of the sand-filled pipe was determined. The onset pressure at each measuring point was determined and the plugging rate in each section of the sand-filling pipe was calculated.

(7) Study of the migration and plugging characteristics of the micropore throat of the gel system: the saturated micro-simulation glass etching model was used to conduct the experiment. The water flooding, gel injection (staining with methyl blue), and the second water flooding step were carried out. Image J was used to process the image, and the distribution characteristics corresponding to the micro-displacement oil and water in different displacement stages were studied and analyzed [30].

## 4. Conclusions

(1) The primary method previously used to enhance the temperature and salt resistance of polyacrylamide polymer involved the introduction of functional monomers into the polymer systems. However, conventional free radical polymerization products are random polymers, and merely introducing functional monomers into the systems cannot effectively address the common problem of inadequate temperature and salt resistance of gels. Theoretical quantification was carried out to quantify the effect of functional monomers and establish a structure–design strategy. Based on this, a controlled active polymerization method was adopted to conduct the polymerization process. The resulting polymer exhibited high regularity, and good control over molecular structure could be achieved. The strength and stability of the polymer could be improved using the crosslinking method and forming a gel. As a result, the temperature and salt resistance of the materials could be improved.

(2) A weak gel based on a hydrophobic associative polymer was formed by crosslinking a hydrophobic associative polymer with a phenolic resin-based crosslinking agent. The concentrations and cohesion ratios of the crosslinking agents were tuned. The optimal polymer concentration was identified to be 2000 mg/L, and the optimal cohesion ratio was 1:1.5. The optimal concentration of the crosslinking agent was 2000 mg/L. The average viscosity retention rate recorded after 90 days of long-term aging was 85.1%. SEM technique was used to observe the micro-morphology of the gel samples at different time periods. The skeleton of the gel network was well maintained in the time spanning 5–90 days. No serious thermal decomposition phenomena, such as curling or depolymerization, were observed in this time frame. The presence of NVP side groups on the chain made the polymer more rigid and stable.

(3) The core flow experiment demonstrated that the hydrophobic associative polymer gel performed well when injected in sand-filled tubes with varying permeability properties. The pressure remained stable at each pressure point, and a good injection performance was achieved. It was observed that after gel plugging, the flow rate of the low permeability layer increased to varying degrees under three different conditions (200, 40, and 5), resulting in a 29.06%, 35.78%, and 48.33% improvement in the utilization degree of the low-permeability layer, respectively. It was also revealed that the pressure could be successfully generated at each measuring point of the sand-filled pipes under different permeability conditions. The plugging rate at each point was greater than 85%, indicating that the weak gel formed from the hydrophobic associative polymer exhibited good injection properties, and the gels could effectively block large pore channels.

(4) The formed gel effectively blocked the high permeability pores along the main stream line. Subsequently, the injection liquid was driven by water to expand the swept volume of the middle- and low-permeability areas and the non-mainstream areas, which resulted in a notable improvement in the extent of oil recovery.

## Figures and Tables

**Table 1:** Viscosity changes recorded for the hydrophobic weak gel system under conditions of varying agent concentrations.

No.	Polymer Concentration (mg/L)	Crosslinking Agent Concentration (mg/L)	Additive Concentration (mg/L)	Viscosity of Gel System at Different Times (mPa·s)						90 Days Viscosity Retention (%)
				Inital	5 Days	10 Days	30 Days	60 Days	90 Days	
A1	2000	3000	1500	15.32	922	987	1198	922	851	86.2
A2	2000	3000	2000	11.05	1225	1582	1802	1465	1239	85.3
A3	2000	3000	2500	18.56	1363	1790	2115	1610	1278	78.4

**Table 2:** Changes in the viscosity of the hydrophobic weak gel system recorded under conditions of varying cohesion ratios.

No.	Polymer Concentration (mg/L)	Crosslinking Agent Concentration (mg/L)	Additive Concentration (mg/L)	Viscosity of Gel System at Different Times (mPa·s)						90 Days Viscosity Retention (%)
				Inital	5 Days	10 Days	30 Days	60 Days	90 Days	
B1	1800	2200	2000	13.08	805	1292	1448	774	659	66.6
B2	1800	2700	2000	11.28	1014	1473	1673	1273	814	65.7
B3	1800	3600	2000	10.60	1000	1462	1617	1129	893	73.6
B4	2000	2400	2000	11.72	1258	1482	1696	1109	1059	80.6
B5	2000	3000	2000	11.05	1226	1584	1803	1465	1239	85.3
B6	2000	4000	2000	10.10	1547	1868	2177	1519	1207	72.8
B7	2250	2700	2000	15.80	1001	1222	1432	1099	864	77.1
B8	2250	3400	2000	16.30	1383	1749	1926	1406	1140	75.4
B9	2250	4500	2000	18.10	1213	1700	1866	1343	977	69.2

**Table 3:** Diversion rates of cores recorded under conditions of varying permeability levels.

Conditions	Permeability Ratio	Permeability (10^−3^ μm^2^)		Flow Rate of High Permeability Layer%		Flow Rate of Low Permeability Layer%		Increase of Flow Rate of Low Permeability Layer %
		High Permeability Layer	Low Permeability Layer	Water Flooding	Sequent Water Flooding	Water Flooding	Sequent Water Flooding	
40,300.86 mg/L, 120 °C	200	1000	5	96.84	67.2	3.16	32.8	29.64
	40	800	20	94.64	58.6	5.36	41.4	36.04
	5	200	40	85.03	36.4	14.97	63.6	48.63

## Data Availability

All relevant data have been presented in this paper.
